# Influence of Perceived Job Demands on Professional Quality of Life and Turnover Intentions of Haematology Nurses: A Cross-Sectional Study

**DOI:** 10.1155/2024/6626516

**Published:** 2024-03-20

**Authors:** Sheng Lian Tan, Huaqiong Zhou, Huimin J. Thian, Phillip R. Della

**Affiliations:** ^1^Nursing Division, Singapore General Hospital, Singapore, Singapore; ^2^Curtin School of Nursing, Curtin University, Perth, Western Australia, Australia

## Abstract

**Background:**

Haematology nurses in Singapore experience a highly stressful work environment and increased workload due to the growing number of patients and complex treatment regimens. High job demands can lead to burnout and high staff turnover rates, which compromises the quality of patient care.

**Aims:**

To assess perceived work demands, levels of social support from colleagues, professional quality of life (ProQOL), and turnover intentions among haematology nurses and examine whether demographic and occupational characteristics, perceived job demands, and support were associated with ProQOL and turnover intentions.

**Design:**

A descriptive, correlational, and cross-sectional design was used in this study.

**Methods:**

A convenience sample of 60 haematology nurses working at a tertiary hospital in Singapore completed a self-administered survey. Perceived job demands, support from colleagues, ProQOL, and turnover intentions were measured using the Copenhagen Psychosocial Questionnaire (COPSOQ III), Professional Quality of Life Scale version 5 (ProQOL5), and Turnover Intention Scale (TIS-6). Descriptive statistics, chi-square test, and multiple linear regressions were employed for data analysis.

**Results:**

Haematology nurses face high cognitive and emotional demands and receive high levels of support from colleagues at work. The majority of the participants reported moderate to high levels of compassion satisfaction (78.3%), burnout (76.7%), and secondary traumatic stress (81.7%). 53.3% of the participants expressed their intention to leave. Perceived job demands were significant predictors of haematology nurses' ProQOL and turnover intentions. Compassion fatigue also significantly predicts turnover intentions.

**Conclusion:**

The high levels of burnout and secondary traumatic stress reported by haematology nurses highlight an urgent need to implement strategies to help nurses cope with the high work demands and reduce their levels of compassion fatigue. *Implications for Nursing Management*. The findings in this study can help nursing leaders understand haematology nurses' perceived job demands and ProQOL, to develop strategies to improve the workplace environment and retention.

## 1. Introduction

The global nursing workforce shortage is a growing issue that has been exacerbated by the COVID-19 pandemic [1]. Nursing workforce shortages are especially significant in the oncology and haematology disciplines [2]. Besides monitoring patients closely for adverse treatment side effects and toxicities, oncology and haematology nurses are also being subjected to highly emotive situations on a regular basis and are at high risk of occupational exposure from chemotherapy administration [2]. The hazardous and highly stressful work environments of cancer care present challenges for organisations to recruit and retain oncology and haematology nurses [2, 3]. In Singapore, the growing numbers of haematology patients coupled with new and complex treatment regimens, such as the chimeric antigen receptor (CAR) T-cell therapy, have increased workload and demands for nurses in the haematology discipline. Retention of experienced haematology nurses is essential to support these demands and maintain quality patient care and optimal patient outcomes [2]. Therefore, gaining an improved understanding of factors that influence work-related outcomes such as professional quality of life and turnover intentions is crucial to retaining nurses in this discipline.

Haematology nurses have frequently been classified as a subset of oncology nursing in published research studies [4, 5]. However, providing clinical care for patients with haematological disorders comes with unique circumstances and challenges. Haematological malignancies have unpredictable disease trajectories, and treatment regimens are often more complex and intensive than those administered for solid tumours [4]. Therefore, haematology patients spend significantly more time in the hospital, especially those who undergo haematopoietic stem cell transplants [5]. The prolonged interaction allows haematology nurses to develop sustained and therapeutic relationships with the patients, which increases nurses' vulnerability to emotional distress and burnout when the patients experience poor outcomes or are potentially at the end of life [4]. Burnout and stress can lead to high staff turnover rates, further aggravating the haematology nursing workforce shortage [2].

Workload or workplace demands have been identified as a primary factor associated with job dissatisfaction, burnout, coping, and intention to leave the discipline among haematology and oncology nurses [6–9]. Studies had revealed that nurses felt guilt or frustration when workload or workplace demands prevented them from spending sufficient time talking to patients or developing a connection with them [10–13]. Nurses deem having ample time with patients to be crucial in the provision of high-quality care and being unable to do so could likely be a precipitating factor for compassion fatigue [10–13]. Workload was found to be the sole independent significant predictor of exhaustion and the single factor that differentiates nurses who were experiencing high levels of exhaustion from those experiencing low to average levels of exhaustion among 230 Australian cancer nurses [7].

Teamwork and support from colleagues were identified as a key resource that helped oncology and haematology nurses cope with challenges at work [10, 12, 14]. Nurses who experienced secondary traumatic stress expressed that social support from their colleagues helped them cope with difficult situations at work and that their colleagues understood them the best [12]. Likewise, early career nurses reported that their coworkers were the only ones who could relate to the workplace frustrations that they experienced, and support from coworkers helped them to buffer stress during work [10]. In addition, teamwork and support from colleagues were found to reduce compassion fatigue and burnout in oncology and haematology nurses [3, 13, 15]. Oncology nurses in the United States and Canada were revealed to be less likely to experience compassion fatigue and burnout and derive more compassion satisfaction when they perceived team cohesiveness at work [15].

Professional quality of life (ProQOL) refers to the quality of life perceived by people who work in a helping profession and comprises compassion satisfaction and compassion fatigue [16]. Compassion satisfaction refers to the positive feelings derived from work, whereas compassion fatigue encompasses burnout and secondary trauma from nursing patients suffering or experiencing traumatic stress [16]. Studies that examined demographic variables such as age, gender, marital status, education, and work-related variables such as years of experience associated with ProQOL reported inconsistent findings [15, 17–19]. Besides that, studies conducted in different countries have also reported varying scores and levels of compassion satisfaction, burnout, and secondary traumatic stress among oncology and haematology nurses.

The mean scores of compassion satisfaction ranged from 31.81 to 42.6 [15, 17–20]. For burnout, the mean scores reported ranged from 21.14 to 28.38 [15, 17–20]. The mean compassion fatigue scores reported by oncology nurses in Portugal and China were 25.82 and 21.39, respectively [17, 19]. Besides ProQOL scores, the levels of compassion satisfaction, burnout, and secondary traumatic stress or compassion fatigue among oncology and haematology nurses based on cutoff scores proposed in the ProQOL manual were reported in some studies. In Korea, approximately 75% to 80% of the participants reported average to high levels of burnout and secondary traumatic stress [18]. Duarte and Pinto-Gouveia [17] described similar findings among participants working in different public hospitals in Portugal. In comparison, 82% to 89% of the nurses in Spain expressed average to high levels of burnout and secondary traumatic stress [21]. Despite more participants reporting higher levels of burnout and secondary traumatic stress, a higher percentage (34.3%) of nurses in Spain reported high compassion satisfaction levels compared to the nurses in Korea (28.1%) [18, 21].

Turnover intentions were found to be associated with ProQOL, work demands, and demographic or occupational characteristics of haematology and oncology nurses. Low compassion satisfaction, high burnout, and high secondary traumatic stress were revealed to be significantly associated with participants' intentions to transfer to another unit [21]. Compassion satisfaction and burnout were also reported to be significant predictors of turnover intention among oncology nurses in the United States [20]. In contrast, Wells-English et al. [20] did not find that secondary traumatic stress significantly predicts turnover intentions. Similarly, turnover intentions were only found to be associated with compassion satisfaction and burnout, not secondary traumatic stress [18]. Besides that, Jang et al. [18] also showed that 51.6% of the 285 Korean oncology nurses surveyed had a turnover intention. Interviews conducted by Saifan et al. [22] revealed that some Jordanian nurses working with cancer patients faced difficulties balancing between work demands and their personal lives, which led to intentions to leave their jobs. In contrast, Giarelli et al. [11] found that 18 out of 20 nurses working in a haematology-oncology unit expressed intention to continue working in oncology nursing within the next five years. However, it is of note that none of the 20 participants reported low levels of compassion satisfaction or high levels of burnout and secondary traumatic stress [11]. 59.8% of the nurses surveyed in Turkey wanted to change their wards and clinical speciality, with workload being cited as one of the main reasons for that intent [8]. Meanwhile, Park and Ahn [23] investigated the correlations between demographic and occupational characteristics and turnover intentions of Korean nurses and found age, job ranking, work experience, type of employment, and place of work were significantly associated with turnover intentions.

Findings from the literature review suggested that workplace demands and support from colleagues were potentially associated with oncology and haematology nurses' ProQOL, burnout, stress, and intention to leave. The Job Demands-Resources (JD-R) model postulates that every occupation involves job demands and resources, which are risk factors linked to job-related stress [24]. Examples of job demands include high patient load or emotionally taxing interactions with patients and family [24]. On the other hand, job resources can be found at the organisational, interpersonal, or task levels and include job security, autonomy, and supervisor or coworker support [24]. The JD-R model proposes that job demands and resources set in motion two separate psychological processes in developing work-related strain and motivation [25]. The first is a health impairment process, whereby jobs with high or chronic job demands such as haematology nursing drain employees' physical and mental resources and result in adverse outcomes, such as compassion fatigue and turnover intentions [24]. The second is a motivational process, in which job resources are assumed to promote work engagement and bring about positive outcomes, including organisational commitment and intention to stay [24].

**Figure 1:**
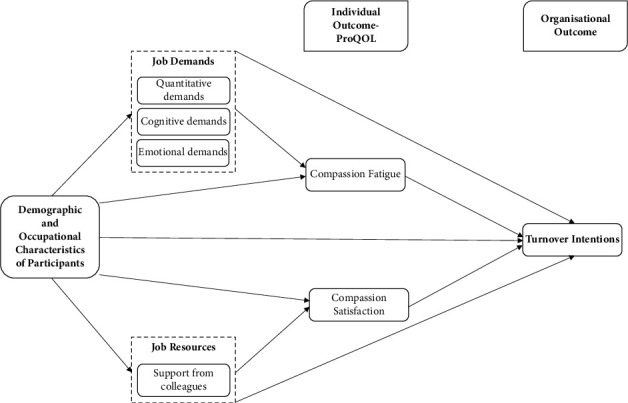
Summary of the constructs and variables measured using the JD-R model.

Inconsistent findings were reported on the associations between demographic and occupational characteristics, job demands, support from colleagues, ProQOL, and turnover intentions of oncology and haematology nurses. Besides that, there is a paucity of research that examines perceived job demands and resources and how these factors influence nurses' ProQOL and turnover intentions, specifically among haematology nurses, as all the studies reviewed considered haematology and oncology nurses as a homogenous group. Therefore, this study aimed to assess the perceived work demands, levels of social support from colleagues, ProQOL, and turnover intentions among haematology nurses in Singapore and examine whether demographic and occupational characteristics, perceived job demands, and support from colleagues were associated with haematology nurses' professional quality of life and turnover intentions. The constructs and variables measured in this study are summarised in Figure 1 using the JD-R model.

## 2. Methods

### 2.1. Research Design and Sample

This study employed a descriptive, correlational design to measure the perceived job demands, support from colleagues, ProQOL, and turnover intentions in haematology nurses and explore the relationships among the variables. Data were collected using a cross-sectional online survey. A convenience sample of registered and enrolled nurses with at least six months of haematology experience working at two inpatient haematology wards at a large tertiary hospital in Singapore was recruited for the study. Advanced practice nurses, resident nurses, and nurses in management roles, including nurse managers and nurse clinicians, were excluded from the study due to differing job scopes and work demands. Nurses on leave during the data collection period were also excluded. 112 nurses met the inclusion criteria. Participants were recruited through two avenues. A recruitment e-mail containing information about the study and a QR code and link to the survey was sent to all eligible nurses who met the inclusion criteria. A follow-up e-mail reminder was sent one week after the initial invitation. In addition, face-to-face information sessions were conducted during daily ward staff meetings for clarifications regarding the study and to enhance participation.

### 2.2. Data Collection

Data were collected over two weeks in October 2021 via Form.gov.sg, a secure self-service online form builder. Participants could review information about the study on the survey web page before answering the questions. Consent was implied when participants submitted a completed survey. Participants were reassured that they could take a break or choose not to complete or submit the survey at any time during the survey process if the questions made them uncomfortable. The survey included demographic and occupational data questions and three instruments to measure workplace demands, support from colleagues, ProQOL, and turnover intentions. Each section was prefaced with introductory information on the variables measured and instructions on selecting the response options. Upon submission of the survey, all responses were stored in an encrypted format on the Form.gov.sg server, and only research team members could view the data.

### 2.3. Outcome Measures and Instruments

In this study, job demands were conceptualised as quantitative, cognitive, and emotional demands. Quantitative demands refer to the number of tasks nurses have to achieve during their work and whether they have sufficient time to complete these tasks in a satisfactory manner [26]. Cognitive demands are tasks or duties requiring the nurses' mental effort [26, 27]. Besides that, haematology nurses experience emotional demands when they manage the emotions of patients and their loved ones at work [26]. Job resources were examined through nurses' perceptions of whether they could obtain support from their colleagues when necessary [26]. ProQOL was chosen as the work-related outcome to investigate haematology nurses' well-being and the development of work-related strain and motivation in this study. Haematology nurses care for patients who go through suffering and highly stressful events and are likely to experience both positive and negative feelings associated with the care they provide. ProQOL provides a balanced perspective of the positive and negative aspects of haematology nursing, including compassion satisfaction and compassion fatigue. This study also examined turnover intentions to determine whether haematology nurses' perceived levels of job demand, support from colleagues, and ProQOL are predictors of their intention to leave.

This study combined three independently validated instruments and specific demographic and occupational data questions to measure the variables of interest. The instruments used included the third version of the Copenhagen Psychosocial Questionnaire (COPSOQ III), the Professional Quality of Life Scale version 5 (ProQOL5), and the 6-item Turnover Intention Scale (TIS-6).

#### 2.3.1. Copenhagen Psychosocial Questionnaire (COPSOQ III)

A short version of the COPSOQ III, comprising 45 items, was used to collect data on perceived job demands and social support received at work. The COPSOQ III is a public instrument. Therefore, the use of the questionnaire did not require consent on the condition that guidelines set out by the COPSOQ International Network were adhered to [28]. The COPSOQ III has been tested for validity and reliability in many countries worldwide and is widely used to assess psychosocial conditions in the healthcare industry [28]. Cronbach's alpha for the psychosocial dimensions under demands at work ranged from 0.77 to 0.80 [26]. For the dimension of social support from colleagues, Cronbach's alpha was reported to be 0.87 [26]. The short version used for this study was adapted based on the COPSOQ International Network guidelines [28]. The survey included mandatory items labelled CORE in the COPSOQ III and was supplemented by items labelled MIDDLE or LONG from the dimensions of interest [28]. Participants were asked to select the most appropriate response for each question on a 5-point Likert scale. Each question was scaled to the intervals 0 to 100, with each response having equal weight [28]. The mean score for each dimension was calculated by adding up the scores of each item under that dimension and obtaining the average [26, 28]. A mean composite score was computed for overall job demands based on the average scores of the three dimensions. Higher scores indicate greater job demands and more social support received on a range of 0 to 100.

#### 2.3.2. Professional Quality of Life Scale (ProQOL5)

The ProQOL5 is commonly used to measure the positive and negative effects experienced by people working in a helping profession, such as nurses, and has three subscales: compassion satisfaction, burnout, and secondary traumatic stress [16]. The burnout and secondary traumatic stress subscales were combined to measure compassion fatigue [16]. The instrument's validity and reliability have been established through extensive research, with Cronbach's alpha for the three subscales ranging between 0.72 and 0.87 [16]. The ProQOL Office at the Center for Victims of Torture provided permission to use the scale for this study.

The ProQOL5 is a 30-item instrument that uses a 5-point Likert scale with responses ranging from *never* to *very often* (1 = never, 5 = very often) [16]. Participants were asked to select the response that best reflected the frequency of each experience in the last 30 days. For this survey, the words in the italicised brackets were changed to *nurse* or *nursing* to help participants read the scale smoothly, as suggested in the ProQOL manual [29]. Each subscale is comprised of ten questions. Compassion satisfaction was measured using questions 3, 6, 12, 16, 18, 20, 22, 24, 27, and 30 [16]. Questions 1, 4, 8, 10, 15, 17, 19, 21, 26, and 29 were used to measure the burnout component [16]. Secondary traumatic stress was measured by the remaining questions [16]. The scores for each subscale were added up, and the mean scores for burnout and secondary traumatic stress were combined to obtain the mean score for compassion fatigue. Cutoff scores for each subscale were also calculated based on a quartile system proposed in the ProQOL manual to identify participants with low, moderate, or high levels of compassion satisfaction, burnout, and secondary traumatic stress [16]. Finally, the scores for each subscale were converted to *t* scores for comparison and further analysis [16].

#### 2.3.3. Turnover Intention Scale (TIS-6)

The 6-item Turnover Intention Scale (TIS-6), adapted from a 15-item scale developed by Roodt [30], was used to measure haematology nurses' intention to leave the organisation [31]. The TIS-6 was developed based on the Theory of Planned Behavior and was determined to be a reliable and valid scale used to measure turnover intentions or predict actual turnover, with a Cronbach's alpha of 0.80 [31]. In addition, exploratory factor analysis was conducted to establish the factorial validity of the scale, with item loadings ranging between 0.73 and 0.81 [31]. Responses to the TIS-6 were measured on a 5-point Likert scale [31]. A total score was obtained by adding all item scores, and the midpoint of the scale, which is 18, was used as a cutoff to indicate turnover intention. The author provided permission to use the TIS-6 for this study.

### 2.4. Ethical Considerations

Ethics approval was obtained from the SingHealth Centralised Institutional Review Board (Reference number: 2021/2564). Participation in this study was entirely voluntary. Eligible nurses were provided with information about the study, study participation, contact information of the investigators, survey sections, and estimated time required to complete the survey in the recruitment e-mail and survey web page. Research team members answered all questions regarding the study and survey. Participants were informed that consent was implied when they submitted a completed survey, but they could not withdraw from the study after their responses were recorded due to the anonymity of the survey. The survey was anonymous to ensure privacy and confidentiality. No personal data that made the participants identifiable were collected. Once submitted, all survey responses were encrypted end-to-end and stored in an encrypted format on the Form.gov.sg server. Data collected were kept confidential. A private key was generated during the survey form creation to ensure that only research team members had access to the responses. Research data were stored according to institution policy.

### 2.5. Data Analysis

All survey responses were exported to Microsoft Excel, where the data were labelled, coded, and checked for missing values. Reverse scoring for specific items in the COPSOQ III and ProQOL5 was performed in Microsoft Excel. The response data were then imported to SPSS v27.0 for analysis. Descriptive statistics were employed to describe and analyse the demographic and occupational characteristics of the participants, perceived job demands, support from colleagues, subscales of ProQOL, and turnover intentions. Statistical tests used included frequency distribution, mean, range, and standard deviation. The chi-square test was utilised to assess for significant associations between turnover intentions and the demographic and occupational characteristics of the participants. Some demographic and occupational characteristics data were regrouped before the test was performed. Next, standard multiple regression was carried out in three parts. The first regression analysis was performed to identify factors that significantly predict perceived job demands and support from colleagues. The subsequent analysis examined the factors predicting ProQOL in the participants. Finally, the last standard multiple regression was used to determine whether perceived job demands, colleague support, and ProQOL were significant predictors of turnover intentions. The assumptions of multiple regression analysis, including multivariate outliers, multicollinearity, normality, linearity, and homoscedasticity of residuals, were assessed and met in all models. A significance level (*p* value) of less than 0.05 was used to determine statistical significance.

## 3. Results

### 3.1. Characteristics of Participants

A total of 60 respondents completed the surveys with a response rate of 54%. Based on the sample size of 60 in this study, power analysis was performed. The power of the test to detect a large effect (Cohen's *f*^2^ = 0.35) was 0.858, a medium effect (Cohen's *f*^2^ = 0.15) was 0.454, and a small effect (Cohen's *f*^2^ = 0.02) was 0.088. Each respondent answered all the survey questions; therefore, all 60 responses were included in the data analysis. Participants' demographic and occupational characteristics are summarised in Table 1. There were 28 participants aged between 21 and 30 (46.7%), and the remaining were between 31 and 60 years of age (*n* = 32, 53.3%). The majority of the participants were female (*n* = 51, 85%). There was an even distribution between participants who were single (*n* = 32, 53.3%) and married (*n* = 28, 46.7%). More than half of the participants do not have children (*n* = 35, 58.3%). With respect to the highest education qualification, 48.3% of the participants held a bachelor's degree (*n* = 29), 25% had a diploma (*n* = 15), and 11.7% had an advanced diploma (*n* = 7). Senior staff nurses and staff nurses represented 40% (*n* = 24) and 36.7% (*n* = 22) of the study sample, respectively. Assistant Nurse Clinicians made up the smallest number of responses (*n* = 3, 5%). Most of the participants (*n* = 53, 88.3%) reported being on rotating shifts. The haematology experience of the participants ranged from 1 to 33 years, with a mean of 9 years and a standard deviation (SD) of 7.65 years. The reported nursing experience of participants ranged from 1 to 40 years, with a mean of 11.99 years and an SD of 9.94 years.

### 3.2. Perceived Job Demands and Social Support from Colleagues

The participants reported a mean job demands score of 56.61 (SD = 12.81). The quantitative, cognitive, and emotional demands scores were 41.46 (SD = 15.27), 69.90 (SD = 14.67), and 58.47 (SD = 20.21), respectively. The mean score for social support from colleagues was 65.42, with a standard deviation of 19.71. Participants reported a maximum score of 100 for cognitive demands, emotional demands, and social support from colleagues. In terms of cognitive demands, 53.3% (*n* = 32) and 58.3% (*n* = 35) of the participants selected “Always” as a response to the questions “Do you have to keep your eyes on lots of things while you work?” and “Does your work require that you remember a lot of things?”, respectively. For emotional demands, 58.3% (*n* = 35) of the respondents indicated that their work was emotionally demanding to a large or very large extent. On the other hand, 21.7% (*n* = 13) of the participants answered “Always” when asked, “How often do you get help and support from your colleagues, if needed?”

### 3.3. Professional Quality of Life

Table 2 presents the ProQOL raw scores and levels reported by the participants. The mean raw compassion satisfaction, burnout, and secondary traumatic stress scores were 36.20 (SD = 7.07), 25.93 (SD = 5.54), and 25.32 (SD = 6.37), respectively. The mean raw compassion fatigue score was 25.63 (SD = 5.40). The cutoff scores for each subscale were calculated based on a quartile system proposed in the ProQOL manual. Majority of the participants reported moderate to high levels of compassion satisfaction (*n* = 47, 78.3%). On the other hand, most of the participants reported moderate to high levels of burnout (*n* = 46, 76.7%) and secondary traumatic stress (*n* = 49, 81.7%).

### 3.4. Turnover Intentions

The mean turnover intention score was 18.32 (SD = 5.00). A cutoff score of 18, based on the midpoint of the TIS-6 scale, was used to determine turnover intentions. A score of 18 or more indicated that the participant had an intention to leave the organisation. More than half of the participants reported an intention to leave the organisation (*n* = 32, 53.3%).

### 3.5. Relationships between Demographic and Occupational Characteristics, Perceived Job Demands, Social Support from Colleagues, Professional Quality of Life, and Turnover Intentions

Initial standard multiple regression models revealed that there is no association between demographic and occupational characteristics and participants' perceived job demands and job resources (all *p* > 0.05). Next, both perceived job demands and job resources were entered as independent variables, together with the demographic and occupational characteristics, in the standard multiple regression analysis to determine their predictive relationships on the ProQOL of participants. Two models were constructed, one each for compassion satisfaction and compassion fatigue.  Table 3 presents the results of the two standard multiple regression models. In the compassion satisfaction model, highest education qualification (*B* = 8.59, *p*=0.001) and job demands (*B* = −0.31, *p*=0.002) were identified to be significant predictors of compassion satisfaction. Education level was a positive predictor of compassion satisfaction, while job demands were negatively associated with compassion satisfaction. The two variables accounted for a significant 48% of the variability in the participants' compassion satisfaction (*R*^2^ = 0.48, *p* ≤ 0.001). In the compassion fatigue model, only job demands were significantly associated with compassion fatigue (*B* = 0.249, *p*=0.015). Job demands significantly accounted for 32% of the variance in compassion fatigue reported by the participants (*R*^2^ = 0.32, *p*=0.009).

Finally, the relationships between the participants' demographic and occupational characteristics, perceived job demands, job resources, ProQOL, and turnover intentions were investigated using the chi-square test and standard multiple regression analysis. The chi-square test showed no significant association between the sample characteristics and turnover intentions (all *p* > 0.05). Therefore, only years of haematology experience was entered into a standard multiple regression analysis with perceived job demands, job resources, compassion satisfaction, and compassion fatigue to estimate the proportion of variance in turnover intentions that can be accounted for by these variables.  Table 4 presents the results of the standard multiple regression analysis. In the constructed model, only job demands (*B* = 0.168, *p* ≤ 0.001) and compassion fatigue (*B* = 0.187, *p*=0.016) were identified to be significant predictors of turnover intentions. Both job demands and compassion fatigue were positively associated with turnover intentions. The two variables accounted for a significant 54% of the variability in turnover intentions of the participants (*R*^2^ = 0.54, *p* ≤ 0.001).

## 4. Discussion

Overall, the participants in this study reported a moderate level of workplace demands (*M* = 56.61, SD = 12.81). The results revealed that the nurses reported a higher level of cognitive (*M* = 69.90, SD = 14.67) and emotional (*M* = 58.47, SD = 20.21) job demands compared to quantitative (*M* = 41.46, SD = 15.27) demands. This finding is consistent with previous studies [6, 9]. More than half of the participants in this study reported that they always must keep an eye on and remember lots of things while working. This reflects the nature of haematology nursing, as the nurses are accountable for administering complex treatment regimens and monitoring patients for treatment side effects and chemotherapy toxicities [4, 6]. Besides that, 58.3% of the participants expressed that their work was emotionally demanding to a large or very large extent. The personal attachment that haematology nurses develop towards the patients and families due to repetitive treatment cycles and witnessing the suffering and death of the patients under their care could be the reasons for the high perceived emotional demands [6, 9]. Therefore, providing haematology nurses with specialised training and frequent updates on the management of patients undergoing complex haematology treatments and support on emotion management may help relieve the cognitive and emotional work demands on haematology nurses [7].

This study indicated that participants received a high level of social support from their colleagues (*M* = 65.42, SD = 19.71). This finding is notable as previous studies have suggested that support from colleagues helped oncology and haematology nurses to cope with difficult situations and stress at work [10, 12, 14]. Besides that, support from colleagues is a critical job resource that could enable haematology nurses to complete their tasks on time, which could explain the lower quantitative job demands reported by the participants in this study [24]. Hence, it is crucial to continue promoting teamwork and peer support among haematology nurses to protect them from the harmful effects of job-related stress and improve work engagement [24].

The compassion satisfaction (*M* = 36.20, SD = 7.07), burnout (*M* = 25.93, SD = 5.54), and secondary traumatic stress (*M* = 25.32, SD = 6.37) scores reported by the participants in this study were within the range reported in the existing literature that examined ProQOL in oncology and haematology nurses [15, 17–20]. However, the mean compassion satisfaction score reported by participants in this study was lower than those reported in Western countries, including the United States, Canada, and Portugal [15, 17, 20]. This finding suggests that haematology nurses in Singapore derive less professional satisfaction from their work than oncology and haematology nurses working in Western countries, which is likely due to the differences in the work environment and cultures between different countries [16]. In contrast, the mean burnout and secondary traumatic stress scores identified in this study are higher than those of most previous studies [15, 17, 19, 20]. Therefore, haematology nurses may experience more exhaustion and stress and are exposed to more traumatic stressful events at work compared to a homogenous population of oncology and haematology nurses [16].

Based on the cutoff scores proposed in the ProQOL manual, most of the participants in this study reported moderate to high levels of compassion satisfaction. However, most of the participants also reported moderate to high levels of burnout and secondary traumatic stress. This finding is congruent with the study conducted by Arimon-Pagès et al. [21]. This suggests that despite being rewarding and meaningful, the positive aspects of haematology nursing do not necessarily negate the burnout and stress experienced by haematology nurses [21]. Therefore, retention strategies should be aimed at both improving the professional well-being of haematology nurses and providing them with job-related and personal skills to perform their work effectively and cope with stressful events at work [10, 17, 21].

Giarelli et al. [11] found that only 2 out of 20 haemato-oncology nurses had intentions to leave oncology nursing. However, the results of this study revealed that 53.3% of the 60 haematology nurses surveyed had an intention to leave the organisation. This finding was similar to the results of previous studies conducted [18, 20, 21]. This high percentage of nurses expressing an intention to leave highlights a concern as turnover intentions are positively related to actual turnover [31]. High staff turnover can, in turn, bring about significant consequences for the organisation, such as workforce shortage and poor quality of care and costs for rehiring and training of nurses [31]. Therefore, nursing leaders must understand and improve factors that influence haematology nurses' turnover intentions to retain them in the organisation and profession.

In terms of factors that predict perceived job demands and support from colleagues, no association was found between the demographic and occupational characteristics of participants and their perceived job demands and job resources. This finding suggests that haematology nurses' perceived job demands and resources are primarily influenced by the work environment and aspects of their job instead of individual characteristics.

Among the demographic and occupational characteristics of the participants, the highest education qualification was found to be the only significant predictor of compassion satisfaction. This finding is supported by previous research conducted [15, 18]. Haematology nurses with higher education qualifications may be better equipped with knowledge of the haematology disease processes and patient management, which allows them to provide better quality care to patients and derive more satisfaction from their work [15]. Hence, organisations should encourage and support nurses in pursuing further education and training to improve their ProQOL.

This study also revealed that age, marital status, and years of experience in nursing and haematology did not predict compassion satisfaction or compassion fatigue. This is different from findings in previous studies in which age [15, 17, 18], marital status [18], and years of nursing and oncology experience [15, 18, 19] were found to be significantly associated with the ProQOL of participants. This could be related to the cultural differences between haematology nurses in Singapore and those overseas. One interesting finding to note is that years of haematology and nursing experience were not predictors of ProQOL. This may indicate that contrary to popular beliefs, experienced haematology nurses neither derive more satisfaction from their work nor are at higher risk for compassion fatigue due to their years of experience than early career haematology nurses. Experienced haematology nurses may have been exposed to more negative effects and stressful events from work compared to early career nurses [15, 17]. However, in their years of experience, they may also have gathered more knowledge and expertise to cope with these difficult situations, which moderates the effect on compassion fatigue [15, 17].

Existing qualitative studies indicated that workplace demands could be a precipitating factor for compassion fatigue as nurses are prevented from having ample time to interact with patients and provide quality care [10–13]. The findings from this study confirm past literature and the health impairment process in the JD-R model, in which job demands were found to be significant predictors of compassion fatigue. Furthermore, this study revealed that job demands were negatively associated with compassion satisfaction. As suggested by previous studies, high job demands could prevent haematology nurses from spending sufficient time with patients and providing quality patient care, as nurses need to complete their tasks satisfactorily and keep their eyes on many things concurrently [10–13]. Besides increasing compassion fatigue, this can also cause haematology nurses to experience incongruence between their expectations and the actual patient care provided, leading to lower compassion satisfaction [15]. Therefore, organisations need to provide adequate support for haematology nurses to cope with the demands at work to increase their professional satisfaction and reduce their experience of compassion fatigue.

Perry et al. [13] revealed that colleague support helped reduce the experience of compassion fatigue in oncology nurses. Similarly, Wu et al. [15] reported that perceptions of team cohesiveness reduced oncology nurses' likelihood of experiencing compassion fatigue and increased the compassion satisfaction derived by the nurses. In contrast, this study found that social support from colleagues was not significantly associated with both compassion satisfaction and compassion fatigue in haematology nurses in Singapore. This finding challenges the second proposition of the JD-R model, which suggests that job resources promote work engagement in employees [25]. Therefore, further studies are warranted to investigate the effects of other job resources, including autonomy, supervisor support, and meaning of work, on haematology nurses' ProQOL.

Finally, this study found that job demands and compassion fatigue were significant predictors of turnover intentions for haematology nurses in Singapore. This finding is well supported by previous literature and the JD-R model [8, 12, 13, 18, 20–22, 25]. Existing studies indicate that job demands have significant effects on oncology and haematology nurses' coping, exhaustion, and satisfaction at work, which could influence their decision to leave the organisation or profession [6–9]. Besides that, haematology nurses might face challenges juggling between demands at work and their personal lives, leading to intentions to leave [22]. Similarly, compassion fatigue can have adverse effects on haematology nurses, including physical and psychological exhaustion, difficulty in establishing and maintaining personal relationships, and negative attitudes towards work [12, 13]. These effects could potentially influence haematology nurses' intention to leave the organisation, discipline, or profession [12, 13]. Hence, in addition to helping haematology nurses cope with job demands, interventions must be taken to reduce burnout and secondary traumatic stress experienced by this group of nurses to retain them in the discipline and organisation.

This study is one of the few that addressed haematology nurses as a heterogenous sample instead of classifying them as a subset of oncology nurses. The study also contributed to understanding how each variable interacts to influence work-related strain, professional satisfaction, and turnover intentions in haematology nurses. Despite that, this study had some design-related limitations that should be noted. Inferences about causal relationships could not be made in this study as a cross-sectional design was used. A second limitation of this study was the sampling bias related to using a convenience sample. The nurses who chose to participate in the study might not represent the population, limiting the generalisability of the study's findings. Using self-administered surveys for data collection can eliminate interviewer bias and allow participants to remain anonymous. However, self-reports are more susceptible to response biases, which might distort the findings. Finally, the small sample size limits the power to detect medium and small effect and generalisability of the study findings. Therefore, it is recommended that the study is replicated with a large random sample from multiple centres or countries to improve the generalisability of the findings. Researchers can also conduct a longitudinal study to examine the long-term effects of job demands on haematology nurses' ProQOL and turnover intentions. Besides that, future research can consider exploring other predictors that influence the above psychosocial factors and outcomes, including personality and resilience of participants, autonomy, and supervisor support.

## 5. Implications for Nursing Management

Nursing leaders and nurse managers can engage nurses in conversations to understand their needs and how the organisation can support them in managing demands at work [11]. Strategies can be developed at organisation level to improve the workplace environment to help nurses cope with work demands and enhance their professional quality of life. For example, organisations can provide education and training for haematology nurses to equip them with the specialised knowledge, skills, and competencies to cope with the demands at work. Training programs can focus on haematology disease processes and patient management, emotion management skills, and building resilience to improve the well-being of haematology nurses and prevent compassion fatigue [21, 32]. Besides that, organisations can adopt technology and informatics, such as wearable devices and smart pumps, to reduce workplace demands placed on haematology nurses. Nursing leaders can also establish formal support mechanisms such as counselling and debriefing sessions for nurses to express their emotions and grief after the loss of a patient to prevent or reduce compassion fatigue in haematology nurses [21, 22]. More emphasis can be placed on mental health of haematology nurses and promoting self-care to cope with the emotional demands at work and reduce the effects of compassion fatigue [11, 12].

## 6. Conclusion

This study revealed that haematology nurses in Singapore face high cognitive and emotional demands at work. Although most of the nurses reported moderate to high compassion satisfaction, the majority of them also experienced moderate to high levels of burnout and secondary traumatic stress. Job demands significantly predict the ProQOL of haematology nurses. Besides that, both job demands and compassion fatigue were significant predictors of turnover intentions in haematology nurses. The high percentage of haematology nurses reporting an intention to leave the organisation in this study highlights an urgent need to implement strategies to help nurses cope with the high work demands and reduce their levels of compassion fatigue, to improve staff retention.

## Figures and Tables

**Table 1:** Demographic and occupational characteristics of participants (*N* = 60).

Characteristics of participants	*n*	%
*Age*		
21–25	13	21.7
26–30	15	25.0
31–35	11	18.3
36–40	5	8.3
41–45	2	3.3
46–50	4	6.7
51–55	7	11.7
56–60	3	5.0

*Gender*		
Female	51	85.0
Male	9	15.0

*Marital status*		
Single	32	53.3
Married	28	46.7

*Have child/children*		
Yes	25	41.7
No	35	58.3

*Highest education*		
Nitec in nursing	5	8.3
Diploma	15	25.0
Advanced diploma	7	11.7
Bachelor's degree	29	48.3
Master's degree	1	1.7
Others	3	5.0

*Job position*		
Enrolled nurse	2	3.3
Senior enrolled nurse	6	10.0
Principle enrolled nurse	3	5.0
Staff nurse	22	36.7
Senior staff nurse	24	40.0
Assistant nurse clinician	3	5.0

*Shift pattern*		
Rotating	53	88.3
Fixed	7	11.7

*Years of working experience*	Mean (SD)	Min–Max
Haematology	9.00 (7.65)	1.0–33.0
Nursing	11.99 (9.94)	1.0–40.0

**Table 2:** Professional quality of life (*N* = 60).

Subscales	*n* (%)	Mean	SD	Min	Max
*Compassion satisfaction*		36.20	7.07	20	50
High	14 (23.3)				
Moderate	33 (55.0)				
Low	13 (21.7)				

*Burnout*		25.93	5.54	13	44
High	12 (20.0)				
Moderate	34 (56.7)				
Low	14 (23.3)				

*Secondary traumatic stress*		25.32	6.37	14	44
High	12 (20.0)				
Moderate	37 (61.7)				
Low	11 (18.3)				

*Compassion fatigue* ^*∗*^		25.63	5.40	15	42

^*∗*^Combined mean score of burnout and secondary traumatic stress. Cutoff scores proposed in the ProQOL manual based on a quartile system. Compassion satisfaction: low = 30 or less; moderate = between 31 and 42; high = 43 or more. Burnout: low = 21 or less; moderate = between 22 and 30; high = 31 and more. Secondary traumatic stress: low = 19 or less; moderate = between 20 and 30; high = 31 or more.

**Table 3:** Standard multiple regression model testing demographic and occupational characteristics, perceived job demands, job resources, and ProQOL.

Model	Independent variable	*B*	SE	*ß*	*p*	*R* ^2^	*P*
Compassion satisfaction						0.481	≤0.001
	Age	2.750	3.598	0.138	0.448		
	Marital status	−1.667	5.318	−0.084	0.755		
	Have children	5.441	4.956	0.270	0.277		
	Highest education	8.586	2.325	0.433	0.001^*∗∗*^		
	Haematology experience	0.140	0.351	0.107	0.691		
	Nursing experience	0.279	0.290	0.277	0.341		
	Job demands	−0.310	0.095	−0.397	0.002^*∗∗*^		
	Job resources	0.051	0.056	0.100	0.371		

Compassion fatigue						0.316	0.009
	Age	−1.890	3.740	−0.105	0.615		
	Marital status	1.880	5.528	0.104	0.735		
	Have children	−2.689	5.152	−0.148	0.604		
	Highest education	−2.600	2.416	−0.145	0.287		
	Haematology experience	0.132	0.365	0.111	0.719		
	Nursing experience	−0.323	0.302	−0.355	0.289		
	Job demands	0.249	0.099	0.352	0.015^*∗*^		
	Job resources	−0.101	0.058	−0.220	0.089		

*B*: unstandardised coefficients; SE: standard error; *ß*: standardised coefficients; *p*: significance levels of variables; *P*: significance levels of model; ^*∗*^*p* < 0.05; ^*∗∗*^*p* < 0.01.

**Table 4:** Standard multiple regression model testing years of haematology experience, perceived job demands, job resources, ProQOL, and turnover intentions.

Model	Independent variable	*B*	SE	*ß*	*p*	*R* ^2^	*P*
Turnover intentions						0.540	≤0.001
	Haematology experience	−0.077	0.068	−0.118	0.258		
	Job demands	0.168	0.043	0.432	≤0.001^*∗∗*^		
	Job resources	0.044	0.026	0.175	0.088		
	Compassion satisfaction	−0.054	0.068	−0.109	0.428		
	Compassion fatigue	0.187	0.076	0.340	0.016^*∗*^		

*B*: unstandardised coefficients; SE: standard error; *ß*: standardised coefficients; *p*: significance levels of variables; *P*: significance levels of model; ^*∗*^*p* < 0.05; ^*∗∗*^*p* < 0.01.

## Data Availability

The data used to support the findings of this study have not been made available due to confidentiality.

## Supplementary Materials

Supplementary Materials STROBE checklist for cross-sectional studies.
